# Suppression of Tumorigenesis: Modulation of Inflammatory Cytokines by Oral Administration of Microencapsulated Probiotic Yogurt Formulation

**DOI:** 10.4061/2010/894972

**Published:** 2010-10-31

**Authors:** Aleksandra Malgorzata Urbanska, Arghya Paul, Jasmine Bhahena, Satya Prakash

**Affiliations:** Biomedical Technology and Cell Therapy Research Laboratory, Departments of Biomedical Engineering and Physiology, Artificial Cells and Organs Research Center, Faculty of Medicine, McGill University, 3775 University Street, Montreal, QC, Canada H3A 2B4

## Abstract

The objective of this study was to examine the ability of a novel microencapsulated probiotic yogurt formulation to suppress the intestinal inflammation. We assessed its anticancer activity by screening interleukin-1, 6, and 12 (IL-1, 6, 12), secretory levels of tumor necrosis factor-alpha (TNF-*α*), interferon-gamma (IFN-*γ*), prostaglandin E_2_  (PGE_2_), and thromboxane B2 in the digesta obtained from the duodenum, jejunum, proximal, and distal segments of the ileum of C57BL/6J-Apc^Min^/J mice. Formulation-receiving animals showed consistently lower proinflammatory cytokines' levels when compared to control group animals receiving empty alginate-poly-L-lysine-alginate (APA) microcapsules suspended in saline. The concentrations of IL-12 found in serum in control and treatment group animals were significant: 46.58 ± 16.96 pg/mL and 158.58 ± 28.56 pg/mL for control and treatment animals, respectively. We determined a significant change in plasma C-reactive protein: 81.04 ± 23.73 ng/mL in control group and 64.21 ± 16.64 ng/mL in treatment group. Western blots showed a 71% downregulation of cyclooxygenase-2 (COX-2) protein in treatment group animals compared to control. These results point to the possibility of using this yogurt formulation in anticancer therapies, in addition to chronic gut diseases such as Crohn's disease, irritable bowel syndrome (IBS), and inflammatory bowel disease (IBD) thanks to its inflammation lowering properties.

## 1. Introduction

The burden of colon cancer in Western countries is overwhelming, amounting to 50 000 deaths per year in USA alone [1]. Much effort is being devoted to the development of effective therapies for this disease as well as to its prevention.

Inflammation plays a major role in pathogenesis of colorectal cancer, and its evaluation is a powerful tool in screening and understanding the key components that lead to this complex disorder. Normally, the intestinal microflora is effectively confined to the lumen by the epithelium. However, intestinal epithelial barrier defects, for instance, disrupted epithelial tight junctions (leaky gut) may contribute to the chronic inflammation as bacteria that translocate through the epithelium may expose submucosal immune cells to inappropriate antigenic stimulation and incite an inflammatory response towards the commensal microflora [2].

The recognition of the compelling association between intestinal inflammation leading to such disorders as Crohn's disease (CD), ulcerative colitis (UC), and colon cancer has led to an abundance of studies investigating the therapeutic potential of altering luminal bacteria using probiotics.

Probiotics are defined as living organisms in food and dietary supplements which, upon ingestion, improve the health of the host beyond their inherent basic nutrition [3]. 

Probiotic bacteria have beneficial effects on the intestinal epithelia both directly and indirectly, including enhanced barrier function, modulation of the mucosal immune system, production of antimicrobials, and alteration of the intestinal microflora [4]. 

The story of probiotics reaches far into the 1850s when Louis Pasteur identified the first probiotic bacteria, *Lactobacillus*, and in 1900 Dr. Henry Tissier was the first person to attempt using bacteria to treat intestinal diseases. By the 1920s,* Lactobacillus acidophilus* was successfully used by the United States to treat diarrhea and constipation. Today, performance and efficacy testing of probiotic bacteria in gastrointestinal disorders, both in animal studies and clinical trials, continues to be of great interest [5, 6]. 

Furthermore, specific strains of bacteria have been implicated in the pathogenesis of colon cancer [7, 8], in particular, *Lactobacillus acidophilus* and *Bifidobacterium longum* have been shown to reduce incidence of colonic tumors and aberrant crypt foci, respectively, in animal models [9, 10]. Although many studies suggest that probiotics are capable of preventing relapse of chronic intestinal inflammation and have beneficial contributions in disorders such as diarrhea, gastroenteritis, irritable bowel syndrome, and inflammatory bowel disease [11, 12], their therapeutic potential has been hampered by inherent limitations in their use, for example, poor survival during gastric transit, concerns regarding the production, cost, storage, and safety. Microencapsulation is a technique which offers protection to live bacteria from the harsh gastrointestinal environment during transit by use of specialized ultrathin semipermeable polymer membranes [13]. It also limits stimulation of the host immune response as well as minimizes risks of systemic infections, the replacement of the normal intestinal flora and gene transfer [14].

There are many tumor markers which can be measured whose measurement or identification is useful in patient diagnosis or clinical management in cancer. However, as no single marker has been established yet as a practical cancer screening tool either in a general healthy population or in most high risk populations, a set of tests needs to be performed in order to draw conclusions. Cyclooxygenase (COX) is an enzyme which catalyzes the first step in the formation of prostaglandins (PGs), the conversion of arachidonic acid to PGH_2_, followed by the metabolism of PGH_2_ to biologically active end-products, PGD_2_, PGE_2_, PGF_*α*2_, PGI_2_, or thromboxane A_2_ (TxA_2_) via specific synthases [15]. Two cyclooxygenase isoforms, COX-1 and COX-2, have been identified. COX-2 is critical for the development of colorectal neoplasia [16]. COX-2 inhibitors can reduce intestinal inflammation leading to tumorigenesis [17] and are therefore used to treat familial adenomatous polyposis (FAP) patients [18] and patients with colorectal cancer [19]. Accumulating evidence has shown that prostaglandin E_2_ (PGE_2_), the main product of cyclooxygenase-2 (COX-2) activity, can promote a number of molecular mechanisms involved in colorectal carcinogenesis [20] in particular tumor cell proliferation and angiogenesis [21–24]. C-reactive protein (CRP) is an acute-phase systemic protein produced primarily in the liver in response to stimulation by interleukin-6 (IL-6) [25]. In addition to studies which show consistency in demonstrating an increased risk of mortality due to inflammation and subsequent cancer development, CRP and IL-6 have been shown to be associated with total and noncardiovascular mortality [26–28].

Tumor necrosis factor-*α* (TNF-*α*) and interleukin 1-*β* (IL1-*β*) seem to play an important role in ulcerative colitis (UC) in relevant experimental models [29] and are linked to colorectal cancers via inducing the expression of vascular endothelial growth factor (VEGF) [30, 31] or causing a significant increase in the release of soluble B7-H3 in colon cancer cell lines [32].

In this study, we investigated the potential of microencapsulated probiotic bacterial cells contained within our yogurt formulation in reducing intestinal inflammation in an animal model for colorectal cancer, C57BL/6J-Apc^*Min*^/J mice. We provide evidence that the daily gavage of the probiotic formulation to mice reduces expression of COX -2 as well as lowers plasma C-reactive protein (CRP) levels. We have quantified the levels of secretory cytokines IL-1*β*, IL-6, IL-12, PGE_2_, TNF-*α*, IFN-*γ*, and Thromboxane B2 in ileal contents.

## 2. Materials and Methods

### 2.1. Chemicals

Sodium alginate (low viscosity), poly-L-lysine hydrobromide (MW = 27,400), and calcium chloride (A.C.S. reagent) were purchased from Sigma-Aldrich, Canada. Difco Lactobacilli MRS AGAR and Difco Lactobacilli MRS BROTH were purchased from Becton, Dickinson and Company Sparks, USA. Liberty plain yogurt 2% M. F. containing bacterial cultures *Streptococcus Thermophilus, Lactobacillus Acidophilus, Bifidobacterium bifidum, *and *Lactobacillus bulgaricus* was procured from a local grocery store. It contained Calories: 110 kcal, fat: 3.5 g, carbs: 9 g, protein: 9 g, vitamin A 6%, calcium 30% vitamin C 4%, and iron 0% per 175 g serving.

### 2.2. Bacteria and Culture Conditions

Bacterial strain of* Lactobacillus acidophilus* no. 314 ((Moro) Hansen and Mocquot deposited as *Bacillus acidophilus* Moro, Designation 43) used in this study was obtained from ATCC (Manassas, VA) and was cultivated and serially propagated in the MRS medium before experimental use. Incubations were performed at 37°C in a Professional Sanyo MCO-18 M Multi-Gas Incubator under anaerobic conditions (1-2% CO_2_, Atmosphere Generation System AnaeroGen; Oxoid Ltd., Hampshire, England). Bacteria were harvested after 20 hours of the 3rd passage for encapsulation.

### 2.3. Microencapsulation Method

The bacterial strains were microencapsulated into alginate-poly-L-lysine-alginate (APA) membranes. All membrane components were filter sterilized through a 0.22 *μ*m Sterivex-GS filter (Millipore, Bedford, MA, USA) prior to use. Grown cultures were centrifuged at 3000 × g for 15 minutes at 25°C, and the supernatant broth was decanted. The pellet of wet cells was weighed and suspended in 0.85% saline, pooled, and slowly added to a gently stirred sterile 3.3% sodium alginate solution (final concentration adjusted to 1.65% with 0.85% saline). The entire procedure was performed under sterile conditions in Microzone Biological Containment Hood (Microzone Corporation, ON, Canada) and all solutions were autoclaved with the exception of poly-L-lysine which was 0.22 *μ*m sterile filtered prior to usage. APA microcapsules were prepared aseptically using an Inotech Encapsulator IER-20 (Inotech Biosystems Intl. Inc., Switzerland). Freshly prepared microcapsules were washed twice with 0.85% saline and stored at 4°C. Parameters for microencapsulation were as follows: gelation time in CaCl_2_—30 minutes, coating time—10 minutes, nozzle diameter—300 *μ*m, vibrational frequency—918 Hz, voltage >1.00 kV, and current 2 amp.

### 2.4. Preparation of Probiotic Formulation

APA microcapsules loaded with *L. acidophilus* bacterial cells were carefully mixed with Liberté plain yogurt 2% M.F. and suspended in sterile 0.85% saline to 80% (vol/vol) final concentration. The bacterial cell count was kept constant at 10^10^ cfu/mL throughout the experiment. Empty APA microcapsules were suspended in 0.85% saline using the same formulation and stored at 4°C until further use.

### 2.5. Animals

Male heterozygous C57BL/6J-Apc^*Min*^/J [33] mice, 5 or 6 weeks old, were obtained from The Jackson Laboratory (Bar Harbor, ME). Multiple intestinal neoplasia (Min) mice are heterozygous for *Apc (*Min/+), a germ-line truncating mutation at codon 850 of the *Apc *gene, and spontaneously develop pretumoric numerous intestinal neoplasms. They are a popular animal model for studies on human colorectal cancer [33]. The animals were housed in the McIntyre Medical Sciences Building Animal Care Facility in a room with a 12-hour light-dark cycle and controlled humidity and temperature. The mice were maintained in a barrier facility. They were allowed sterile water and the laboratory rodent diet 5001 from Purina Land O'Lakes *ad libitum*. Overall health of the animals was monitored daily. The animal use protocol was approved by the Animal Care Committee of McGill University, and animals were cared for in accord with the Canadian Council on Animal Care (CCAC) guidelines.

### 2.6. Experimental Design

Upon arrival, animals were kept in a sterile environment in individual ventilated cages (IVCs) which filter the air with HEPA filters. The cages, food, water bottles, and so forth were autoclaved. Animals were randomly placed in the cages and allowed one week of acclimatization. The animals were ranked and assigned to groups according to a randomized block design. The mice were separated into two experimental groups: control (*n* = 24) animals were gavaged 0.3 mL of 0.85% saline solution and treatment animals (*n* = 24) were gavaged 0.3 mL of APA microencapsulated *L. acidophilus* bacterial cells blended in 2% M.F. yogurt for the total of 0.66 × 10^10^ cfu/mL of encapsulated bacterial cells per mouse per day. The caloric content of each gavage (0.125 kcal) was considered to be an insignificant factor in potential animal weight gain and therefore not taken into account. Animals were weighed individually every week, and their food consumption was weighed per cage of 4 animals. Blood collection from the saphenous vein was performed every 4 weeks. Blood was separated using 5000 × g at 4°C for 10 minutes.

### 2.7. Luminal Digesta

At the time of sacrifice, the small intestine of each animal was measured and cut into 4 equal segments, each approximately representing a distinctive part, namely, duodenum, jejunum, and proximal and distal ileum. Each segment was flushed with cold D-PBS buffer (Gibco), its contents were collected and flash frozen at −85°C. Before analyses, the digesta samples were thawed and treated with 1.0% BSA-50 mM Tris buffer (pH 7.5) for 60 min at room temperature to separate the food matrix from cellular material. The samples were then centrifuged at 50,000 × g for 15 min. The supernatants were stored at −85°C and used later for measurements.

## 3. Analytical Methods

### 3.1. Quantification of IL-1*β*, IL-6, IL-12, PGE_2_, Thromboxane B2, TNF-*α*, IFN-*γ*, and CRP Expressions Using ELISA

IL-1*β*, IL-6, and IL-12 were quantified using kits from Biosource, Invitrogen, USA according to manufacturer's recommendations. PGE_2_ was measured using a competitive enzyme immunoassay (Cayman Chemical, Ann Arbor, MI) as described previously in [34]. Briefly, 96-well plates were precoated with the capture Ab (goat antimouse Ab). 100 *μ*L enzyme immunoassay (EIA) buffer was loaded to nonspecific binding (NSB) wells. 50 *μ*L EIA buffer was loaded to maximum binding (B_0_) wells. 50 *μ*L PGE_2_  standards were loaded into appropriate wells. 50 *μ*L of samples or standards were incubated with 50 *μ*L of PGE_2_ tracer and 50 *μ*L of PGE_2_ mAb overnight at 4°C. After three washes in wash buffer, 200 *μ*L of Ellman's reagent was added to the plate and allowed to incubate for 1 h for the color to develop. The optical Density (OD) was determined using the Perkin Elmer Victor microtiter plate reader at 405 nm, and PGE_2_ production was expressed as picograms per milligram. 

Thromboxane B2 (Express EIA kit-monoclonal, Cat.No. 10004023, Cayman Chemical, Ann Arbor, MI) was measured according to manufacturer's instructions. TNF-*α* was measured using a competitive enzyme immunoassay (Cat.No. KMC3012, Immunoassay Kit, Biosource Int., Inc, USA) according to manufacturer's instructions. Biotin gamma rabbit antimouse interferon-*γ* was purchased from Cedarlane, (Hornby, ON, Canada) and reconstituted from sterile form to 50 *μ*g/mL with PBS solution containing 0.1% BSA. Murine IFN-*γ*  ELISA kit was purchased from PeproTech (Rocky Hill, NJ) and used as recommended by the manufacturer. CRP was measured in plasma using a mouse CRP ELISA kit (Life Diagnostics, Inc., USA).

### 3.2. Immunoblotting of COX-2

Intestinal tissue samples (flushed with cold PBS) were flash frozen in liquid nitrogen before storing at −85°C. Frozen samples were weighed, and 3 mL of RIPA buffer (Santa Cruz Biotech, CA) (with PMSF in DMSO, protease inhibitor cocktail, and sodium orthovanadate) was added per gram of tissue. The samples were homogenized, pooled, sonicated, and centrifuged at 4°C for 10 minutes at 10,000 × g. The protein content was determined using Quant-iT protein assay kit (Invitrogen, Burlington, Canada) with bovine serum albumin (BSA) as the standard. Twenty micrograms of total proteins, as evaluated by Quant-iT protein assay, from tissue were used for Western blot. Aliquots containing protein were fractionated on 4–12% Bis-Tris Gel (Invitrogen, Carlsbad, CA) at 120 V for 2 hours. After electrophoresis, proteins were transferred from the gel to a nitrocellulose membrane (Whatman, Maidstone, Kent, UK) using Novex Semi-Dry Blotter (Invitrogen, Carlsbad, CA). COX-2 mouse monoclonal antibody (1 : 1,000; Santa Cruz Biotechnology, Santa Cruz, CA) was used as the primary antibody. Horseradish peroxidase-conjugated goat antimouse IgG was used as the secondary antibody (1 : 1,000; Santa Cruz Biotechnology, Santa Cruz, CA). The membrane was then developed with chemiluminescent agents (ECL, BM Chemiluminescence Blotting Substrate (POD), Roche Diagnostics, IN) and visualized in a Versa Doc Imaging System using software Quantity One-4.5.1 (Model 5000, Bio-Rad Laboratories (UK)). Western blot images were analyzed using Image J software (http://rsb.info.nih.gov/ij/ (accessed in December 2005, NIH, USA)).

### 3.3. Adenoma Classification and Enumeration

The number of adenoma, low-grade dysplasia, high-grade dysplasia, and gastrointestinal intraepithelial neoplasias (GIN) was scored by a blinded veterinary pathologist to the treatment in the small and large intestine. The standards for the histological assessment were established from the MMHCC-sponsored symposium and are detailed on the MMHC web site (http://emice.nci.nih.gov/emice/mouse_models/organ_models/gastro_models/murine_intestinal_ neoplasia/models_colorectal_cancer).

### 3.4. Statistical Analyses

All results in this paper are means calculated using Excel and expressed as means ± SEM or SD. Student *t*-test was used to assess the statistical significance of the differences between test and control groups. Data was considered significant at *P* < .05.

## 4. Results

Artificial cell microcapsules containing *L. acidophilus* were prepared using the multistep preparation methods described and were stored at 4°C for use in experiments. Sterile conditions and procedures were strictly adhered during the process of microencapsulation. Results show that the bacterial cells were able to survive the encapsulation process and grow normally when obtained supernatant was plated after breaking of the microcapsule membrane. The microcapsules contained, on average, 10^10^ cfu/mL of bacteria. Freshly prepared microcapsules were spherical and opaque on account of bacterial density. Morphological studies by microscopic analysis revealed that the mean capsule diameter was 433 ± 67 *μ*m, and they exhibited high homogeneity. Bacterial cells were able to survive during the encapsulation process and grow normally (data not shown).

### 4.1. Food Intake and Body Weights

**Figure 1:**
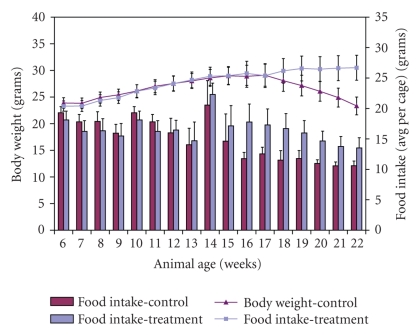
The effect of daily gavage of microencapsulated *L. acidophilus* cells in 2% M.F. yogurt in *Apc*Min mice on animal body weights and food intake (weekly food intake averaged per cage (4 animals)). Data represent the mean ± SEM per group; *n* = 24.

Results show (Figure 1) that all animals gained weight steadily up to week 16 of age in control group (25 ± 1.1 g) and in treatment group (25 ± 1.2 g). All the animals maintained constant weight until 17 weeks of age after which the weight in control group animals decreased to 20 ± 1.2 g whereas treatment group animals continued to increase in their weight up to 27 ± 1.2 g at the time of sacrifice. The food consumption was consistent with body weight gain and loss. Every week after week 14 of age, mice in control group had a lower consumption than treatment group animals. The potential weight gain from the caloric intake originated from yogurt fat was not taken into account due to its insignificant value of (0.125 kcal) per gavage.

### 4.2. Serum IL-12

**Figure 2:**
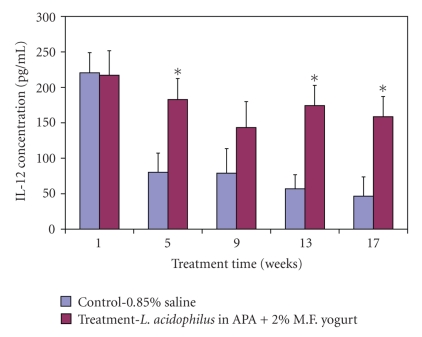
The effect of treatment on IL-12 concentrations in serum. Data represent the mean ± SD of concentration levels per group; *n* = 24. Asterisks: statistical differences (*P* < .05) when compared to control.

During the 17-week experimental period animal serum was used to measure the levels of inflammatory interleukin 12. Results show the concentration levels were significantly higher (weeks 5, 13, and 17, *P* < .05) in treatment animal group compared to control group animals (Figure 2). At the time of sacrifice the average levels were 46.58 ± 16.96 pg/mL and 158.58 ± 28.56 pg/mL for control and treatment animals, respectively.

### 4.3. Luminal IL-12

**Figure 3:**
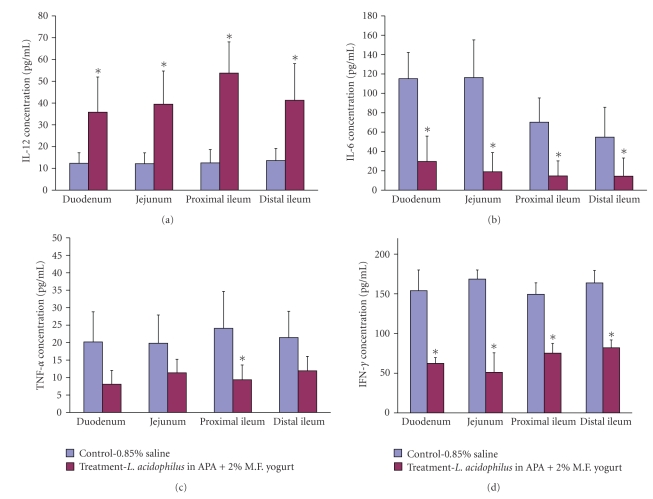
The effect of treatment on luminal cytokine concentration levels found in duodenum, jejunum, and proximal and distal ileum: IL-12 (a), IL-6 (b), TNF-*α* (c), and IFN-*γ* (d). Data represent the mean ± SD of concentration levels per group; *n* = 24. Asterisks: statistical differences (*P* < .05) when compared to control.

Concentrations of luminal IL-12 were measured in 4 distinct parts of the small intestine: duodenum, jejunum, and proximal and distal ileum. In the control group similar concentrations were found in all 4 intestinal sections (12.35 ± 5.55 pg/mL) (Figure 3(a)). Among the treatment group, luminal IL-12 concentrations were the lowest in the duodenum, 35.79 ± 16.13 pg/mL, and the highest in proximal ileum, 53.74 ± 14.29 pg/mL. All measurements were significant when compared to control (*P* < .05).

### 4.4. Luminal IL-6

The concentration of luminal IL-6 was measured in the same sections of the small intestine as IL-12. Control group animals had statistically higher levels of IL-6 in all intestinal sections when compared to treatment group animals (*P* < .05). The IL-6 concentration was especially high in the duodenum and jejunum of control group animals 115.07 ± 27.12 pg/mL and 116.29 ± 38.92 pg/mL, respectively (Figure 3(b)).

### 4.5. Luminal TNF-*α*

The concentration of luminal TNF-*α* was measured in the same manner as described for IL-6 and IL-12. Higher concentrations were detected in control group animals and were relatively comparable in all intestinal sections (Figure 3(c)). The highest concentration was in proximal ileum, 24.08 ± 10.59 pg/mL. The concentrations of TNF-*α* in proximal ileum in treatment group animals were statistically the lowest, 9.38 ± 4.23 pg/mL.

### 4.6. Luminal IFN-*γ*

There were significantly higher concentrations of luminal IFN-*γ* in control group animals when compared to those of the treatment group (Figure 3(d)). The highest concentration of luminal IFN-*γ* was found in the jejunum of the treatment group, 168.55 ± 11.55 pg/mL. The lowest concentration was found in the jejunum of control group animals, 51.08 ± 24.59 pg/mL.

### 4.7. Luminal IL-1*β*

**Figure 4:**
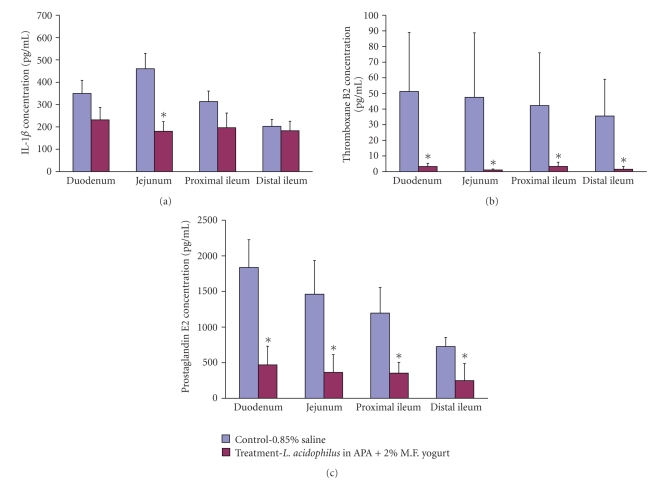
Luminal IL-1*β* (a), thromboxane B2 (b) and prostaglandin E_2_  (c) concentration levels found in duodenum, jejunum, and proximal and distal ileum. Data represent the mean ± SD of concentration per group; *n* = 24. Asterisks: statistical differences (*P* < .05) when compared to control.

The difference in concentration of luminal IL-1*β* was statistically significant in jejunum, *P* < .05 (Figure 4(a)). In control group animals it was found to be 460.4 ± 68.45 pg/mL whereas in the treatment group it was found to be 180.09 ± 43.56 pg/mL. In the duodenum, proximal and distal ileum the concentration levels did not differ statistically from control.

### 4.8. Luminal Thromboxane B2

The thromboxane B2 concentration was especially high in all intestinal sections in control animal group, and its range was from 51.27 ± 23.53 pg/mL in duodenum to 35.50 ± 13.16 pg/mL in distal ileum (Figure 4(b)). On the contrary, the levels of thromboxane B2 found in treatment animal group were relatively low, ranging from 3.31 ± 1.75 pg/mL to 1.52 ± 0.62 pg/mL. All the concentration levels between groups in each intestinal section were statistically significant (*P* < .05).

### 4.9. Luminal PGE_2_

The concentration of luminal PGE_2_ was the highest in control animals in all intestinal sections, especially in duodenum 1836.55 ± 389.88 pg/mL (Figure 4(c)). The concentration of PGE_2_ correlated positively with the total number of adenomas, adenoma burden, and the relative proportion of medium-sized and large adenomas. PGE_2_ correlated negatively with the relative proportion of small adenomas in treatment group. The lowest concentration of PGE_2_  was found in treatment group in distal ileum 248.57 ± 126.88 pg/mL.

### 4.10. C-Reactive Protein (CRP)

**Figure 5:**
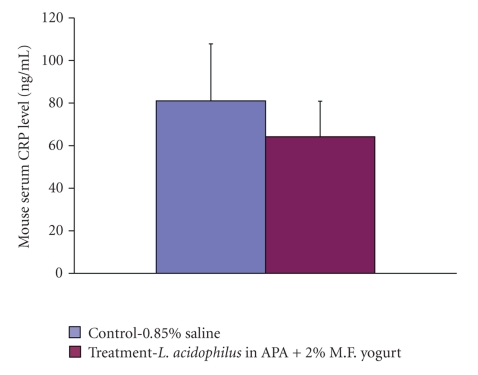
Comparison of plasma C-reactive protein (CRP) levels between control and treatment mice measured by enzyme-linked immunosorbent assay at the time of sacrifice; *n* = 24; error bars represent SD. Asterisks: statistical differences (*P* < .05) when compared to control.

C-reactive protein concentrations were measured using ELISA kit from plasma stored at −85°C obtained from animals by cardiac puncture at the time of sacrifice. The levels between control and treatment group animals were not significant (Figure 5). They were found to be 81.04 ± 23.73 ng/mL in control group and 64.21 ± 16.64 ng/mL in treatment group.

### 4.11. COX-2 Expression

**Figure 6:**
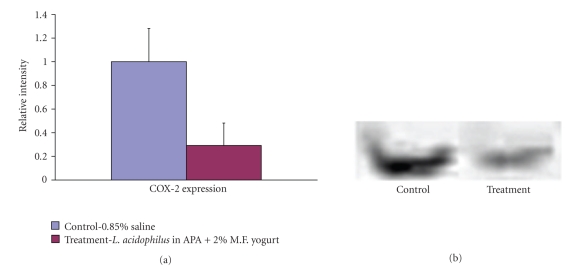
Western blot showing COX-2 expression found in homogenized distal ileum of small intestine tissues in control and treatment, animals, MW of COX-2-72 kD. Samples were pooled per treatment and the average expression was obtained for the total of 24 per group. The relative band intensities were calculated with Image J software (*P* = .018).

Intestinal lysates obtained from distal ileum were analyzed by Western blotting employing antibodies specific for the COX-2 isoform.  Figure 6 shows representative Western Blot bands of the 72 kD COX-2 protein. A higher expression level of COX-2 was found in control group animals. Using Image J software, the bands were analyzed and relative intensities for control and treatment group animals measured. It was found that the COX-2 in treatment group animals was 71% lower than that in control group animals.

### 4.12. Adenoma Classification and Enumeration

There were on average 4.5 ± 1.46 tumors found per animal in control group and 2.5 ± 1.60 tumors in treatment group. Most found lesions were small GIN: a total of 66 in small intestine of control group and 42 in large intestine of treatment-receiving animals. In general, adenomas found in colon of control group (4) and treatment group (2) animals were less numerous. This is 44% decrease in total number of lesions in treatment-receiving animals when compared to control group animals.

## 5. Discussion

Cancer is a chronic pathologic process. Inflammation is considered to be a particularly important factor in the pathogenesis of the colorectal cancers. A period of time is required for a cancer to develop, invade or metastasize, and eventually kill the host. Diet and disease development are strongly related in disease incidence. For instance, the so-called “Western diet”, containing red meat, is considered to be the leading cause of higher colorectal cancer development. The main objective of this study was to suppress the occurrence of spontaneously developing polyps in animals ensuring that the levels of inflammatory cytokines are as low as possible. The immunoenhancing effect of microencapsulated probiotic bacterial cells may be an important mechanism that reduces the growth of malignant tumors. Thus the animals were administered with microencapsulated probiotic bacterial cells daily. The assessment of animal health was achieved by measuring the various inflammation biomarkers at the time of sacrifice. Animals which received daily treatment with probiotic yogurt formulation were able not only to maintain their body weight but also to slightly increase it, which is a general indication of an overall health. They also had a higher food intake comparing to control animals. This may further imply that the rate of disease progression was slower.

Biomarkers are very beneficial to identify pathological processes before individuals become symptomatic or to identify individuals who are susceptible to cancer [35]. Luminal digesta obtained from the intestines were used to give an indication of IL-1*β*, IL-6, IL-12, PGE_2_, Thromboxane B2, TNF-*α*, IFN-*γ*, and CRP levels in the gastrointestinal tract and CRP in the plasma at the time of sacrifice. Our results showed an overall trend indicating notably lower inflammation in the small intestines in the animals receiving daily treatment. As *Apc*Min mice develop spontaneous neoplasia predominantly in the small intestine as opposed to the colon, this study investigated and validated the inflammatory state of that organ. In the intestine, IL-1 has been shown to be an important inflammatory mediator whose levels are increased in inflammatory bowel disease [36, 37]. IL-1*β* is solely active in its secreted form whereas IL-1*α* is mainly active in cell-associated forms (intracellular precursor and membrane-bound IL-1*α*). IL-1*α* is only rarely secreted in a limited manner [38]. The assay showed higher concentrations of IL-1*β* in all sections of the small intestine in control group animals. In addition, IL-6 lower concentration and IL-12 higher levels were indicative of a slower progress of the disease in treatment group animals. Similar results were obtained for TNF-*α* and IFN-*γ*.

These findings are consistent with those from previous studies [39], which further proves that inflammation plays an important role in the development of cancer. Tumor invasiveness can be assessed by measuring thromboxane B2 and PGE_2_. Therefore, we compared the levels of thromboxane B2 and PGE_2_ in the small intestines. PGE_2_ is one of the primary prostaglandins formed from the coupled metabolism of arachidonic acid by the COX-1 and COX-2 and PGE synthases (microsomal and/or cytosolic). Moreover, its activity influences inflammation, fertility and parturition, gastric mucosal integrity, and immune modulation [40, 41]. Accumulating evidence suggests that PGE_2_ has direct effects in enhancing colonic epithelial cell survival by stimulating cell proliferation and survival, tumor cells invasiveness, and production of angiogenic agents [42]. Higher concentrations of PGE_2_ in control group animals further show intestinal inflammation of a greater extent when compared to treatment group animals. As it was postulated before, the inducible cyclooxygenase isoenzyme, COX-2, is significantly overexpressed at sites of inflammation and in various malignant tissues, with concomitant overproduction of the major arachidonate metabolite, PGE_2_ [43].

Increased expression of CRPs has been described in several different malignancies, including colorectal [44], gastric [45], lung [46], renal [47], and breast [48] cancers. We have measured plasma concentrations of C-reactive protein from animals at the time of sacrifice. Elevated levels of CRP were detected in control group animals when compared to animals receiving treatment. C-reactive protein remains significantly associated with a higher risk of colon cancer in *Apc*Min mice. Nonetheless, CRP is a nonspecific marker of inflammation, and additional studies of specific cytokines or factors that regulate acute-phase response are necessary to elucidate the mechanisms by which inflammation increases the risk of colon cancer.

COX-2 expression has a large impact on adenoma growth in *Apc*Min mice, where treatment with a COX-2-specific inhibitor is known to markedly reduce both the numbers and growth of adenomas [49]. Marked upregulation of COX-2 occurs in various cells including endothelial cells during stress and in inflammatory conditions such as sepsis. As COX-2 expression is induced by a number of cytokines including TNF-*α* and IL-1, mitogens or growth factors, lipopolysaccharide (LPS), and other inflammatory stimuli, it was of crucial importance to verify its expression levels. COX-2 levels were different between control and treatment groups, (we obtained 71% reduction of averaged inflammation level in treatment-receiving animals) as expected, further providing evidence that the animals in treatment group have lower intestinal inflammation when compared to control group animals. 

As gastrointestinal intraepithelial neoplasias (GINs) are the precursors of adenomas and later of intestinal carcinoma, it was interesting to note the highest polyp counts of these lesions. Although *Apc*Min mice provide a genetically valid model for studying and understanding intestinal tumorigenesis, its major drawback is that it differs from the cancer development in humans. For instance, in human, carcinogenesis is a complex multistep, often including metastasis process. The polyps found in *Apc*Min mice do not undergo the process of metastasis [50]. Furthermore, adenomas in *Apc*Min mice occur primarily in the small intestine whereas tumors in human are generally restricted to the colon and rectum. To improve the effectiveness of polyp enumeration and classification, one needs to design an automated system (protocol) that would allow consistency by implementing universal guidelines and standards. 

Several factors should be considered in the interpretation of our findings. A major strength of the current study is that it is a prospective study, and, thus, we can more confidently infer a temporal association between inflammation and the occurrence of colon cancer. Compared to other studies [51–53], we obtained greater reduction of inflammation in *Apc*Min mice due to oral treatment with formulation of microencapsulated probiotic bacterial cells and yogurt. Based on the results of the present study, it is not possible however, to explain the tumorigenesis in the *Apc*Min mouse model by immunological responses. Although confirmation of these results is clearly warranted, this finding, if true, could have implications for prevention strategies. Additional studies are needed to clarify the mechanism of bacterial activity and its impact on immunomodulating gastrointestinal tract.

## 6. Conclusions

In conclusion, daily oral administration of the microencapsulated probiotic formulation results in an overall decrease in total number of intestinal lesions and general functioning which leads to increasing host protection against various pathologies. This study supports the role for supplemental probiotics as a strategy both for suppressing inflammation and for preventing colon cancer.
