# Erratum to “Suppression of Tumorigenesis: Modulation of Inflammatory Cytokines by Oral Administration of Microencapsulated Probiotic Yogurt Formulation”

**DOI:** 10.4061/2011/891015

**Published:** 2011-11-30

**Authors:** Aleksandra Malgorzata Urbanska, Arghya Paul, Jasmine Bhathena, Satya Prakash

**Affiliations:** Biomedical Technology and Cell Therapy Research Laboratory, Departments of Biomedical Engineering and Physiology, Artificial Cells and Organs Research Center, Faculty of Medicine, McGill University, 3775 University Street, Montreal, QC, Canada H3A 2B4

In regard to the following paper published in the October 2010 issue of the International Journal of Inflammation: Aleksandra Malgorzata Urbanska, Arghya Paul, Jasmine Bhahena, and Satya Prakash. Suppression of Tumorigenesis: Modulation of Inflammatory Cytokines by Oral Administration of Microencapsulated Probiotic Yogurt Formulation, one of the authors' names was misspelled. We would like to correct the author's name as it appears in the paper from “Jasmine Bhahena” to “Jasmine Bhathena.”

